# Clinical Validity, Understandability, and Actionability of Online Cardiovascular Disease Risk Calculators: Systematic Review

**DOI:** 10.2196/jmir.8538

**Published:** 2018-02-01

**Authors:** Carissa Bonner, Michael Anthony Fajardo, Samuel Hui, Renee Stubbs, Lyndal Trevena

**Affiliations:** ^1^ School of Public Health The University of Sydney Camperdown Australia; ^2^ Ask, Share, Know: Rapid Evidence for General Practice Decisions Centre for Research Excellence Discipline of General Practice The University of Sydney Camperdown Australia

**Keywords:** cardiovascular disease, risk assessment, risk communication, risk formats

## Abstract

**Background:**

Online health information is particularly important for cardiovascular disease (CVD) prevention, where lifestyle changes are recommended until risk becomes high enough to warrant pharmacological intervention. Online information is abundant, but the quality is often poor and many people do not have adequate health literacy to access, understand, and use it effectively.

**Objective:**

This project aimed to review and evaluate the suitability of online CVD risk calculators for use by low health literate consumers in terms of clinical validity, understandability, and actionability.

**Methods:**

This systematic review of public websites from August to November 2016 used evaluation of clinical validity based on a high-risk patient profile and assessment of understandability and actionability using Patient Education Material Evaluation Tool for Print Materials.

**Results:**

A total of 67 unique webpages and 73 unique CVD risk calculators were identified. The same high-risk patient profile produced widely variable CVD risk estimates, ranging from as little as 3% to as high as a 43% risk of a CVD event over the next 10 years. One-quarter (25%) of risk calculators did not specify what model these estimates were based on. The most common clinical model was Framingham (44%), and most calculators (77%) provided a 10-year CVD risk estimate. The calculators scored moderately on understandability (mean score 64%) and poorly on actionability (mean score 19%). The absolute percentage risk was stated in most (but not all) calculators (79%), and only 18% included graphical formats consistent with recommended risk communication guidelines.

**Conclusions:**

There is a plethora of online CVD risk calculators available, but they are not readily understandable and their actionability is poor. Entering the same clinical information produces widely varying results with little explanation. Developers need to address actionability as well as clinical validity and understandability to improve usefulness to consumers with low health literacy.

## Introduction

Online health information may be the first step toward seeking professional medical advice, so the quality of this information is important: is it clinically valid, does it communicate risk effectively, is it understandable to the user, and what actions does it prompt? Unfortunately, the majority of users may not have the necessary skills to effectively evaluate these issues. Health literacy is the ability to access, understand, and make use of health information and services [1], and a large proportion of the general population has inadequate skills [2] (poor health literacy skills in Australia: 59% [3], Europe: 47% [4], Canada: 60% [5]). Low health literacy is associated with less trust in online health information, decreased ability to evaluate that information, and worse health outcomes for cardiovascular disease (CVD) and other chronic conditions requiring self-management [2,6,7]. The issue of eHealth literacy is a related but separate barrier to using health information online—not only do users need to understand and act on the information, but they need the basic skills to find reliable websites in the first place [8]. Less educated, low income, and older individuals may be particularly disadvantaged by inaccessible and poorly explained online health information [2,6,9]. While there is no consensus on how best to evaluate health websites, the majority of studies have concluded that the quality is low [10]. EHealth interventions are increasingly common but have largely neglected the issue of health literacy, which may contribute to their low use [7,8,11].

CVD is the leading cause of mortality and morbidity worldwide, but its incidence can be reduced through risk factor modification via lifestyle change and/or medication [12-15]. This makes it a highly relevant issue for eHealth, as it affects a large proportion of the population and may be prevented through individual behavior change before medical intervention is necessary. Many eHealth interventions target lifestyle with the ultimate aim of preventing CVD [11], but how do individuals know when to access professional medical assistance? The decision to prescribe medication should be based on the likelihood of avoiding a heart attack or stroke, which depends on the baseline absolute CVD risk for an individual [16]. CVD prevention guidelines often use algorithms based on large cohort studies to estimate the risk of a CVD event, usually over 5 or 10 years [12,13]. For example, by identifying 1000 patients with an absolute CVD risk of >21% and lowering their blood pressure, 38 heart attacks and/or strokes would be prevented over 5 years [17,18]. On the other hand, identifying 1000 patients with an absolute CVD risk <11% and treating their blood pressure would prevent only 14 CVD events over 5 years [17,18]. Both groups would however, be exposed to the potential side effects, costs, and inconvenience of antihypertensive medication in order to achieve these reductions in CVD event rates [18]. To estimate the absolute risk of a CVD event, numerous tools exist using different parameters and models [19]. The commonly used Framingham model is based on age, sex, smoking, diabetes, cholesterol, and blood pressure [20]. More recent models used in UK and US guidelines include ethnicity and socioeconomic indicators [13,15]. These risk calculators are available to the public online, but little is known about their quality.

Previous research indicates that online CVD risk calculators can be easily misunderstood. Users may enter their risk factors incorrectly, the provision of multiple risk formats can be confusing if not explicitly explained, and the risk calculators themselves may make assumptions about missing data that lead to less accurate results [21,22]. Users may also question the credibility of the calculator’s results if their prior expectations are not met [23]. On the other hand, an engaging interactive format can increase the emotional response to the risk result and potentially motivate action more than a standard verbal description of risk by a doctor [24,25].

This study aimed to systematically review publicly available online risk calculators for CVD and evaluate them on criteria relevant to health literacy (clinical validity, risk communication, understandability, and actionability).

## Methods

### Procedures

The general approach for this study was to follow a systematic review process using 2 independent searchers (SH, RS) who qualitatively described each risk calculator and evaluated them quantitatively based on the validated Patient Education Material Evaluation Tool for Print Materials (PEMAT-P) scale. For the search and evaluation, we used a third rater (MF) to resolve discrepancies and reach consensus in accordance with section 7.6 of the Cochrane Handbook for Systematic Review of Interventions [26]. The qualitative descriptions of the risk calculator were used to develop a framework for quantitative data extraction (risk model, risk result, and presence/absence of risk communication formats), after which an individual researcher (MF) conducted the basic data extraction. This process was discussed and refined with the lead researcher (CB) on a fortnightly basis, with additional advice from a general practitioner/academic researcher (LT).

### Ethical Approval

Since there were no participants in this study and the data was based on publicly available websites, an ethics application was not required.

### Inclusion and Exclusion Criteria

Online risk calculators were considered if they met the following inclusion criteria: (1) assessed risk of future CVD in individuals without a previous CVD event, (2) were available without the need for registration or payment, and (3) were interactive. Calculators were not considered if they were downloadable files such as an Excel (Microsoft Corp) spreadsheet or PDF, addressed absolute risk of future cardiovascular events in people with atrial fibrillation, or did not provide a risk result for the end user.

### Search Strategy

There were 2 main search strategies for identifying Web addresses that contained the CVD risk calculators. The first strategy was to access predetermined reputable websites including 6 national heart foundation websites (Australian National Vascular Disease Prevention Alliance, the National Heart Foundation of New Zealand, the Joint British Societies, the UK National Health Service, the American Heart Association, and the American College of Cardiology) and a not-for-profit source [27], and the second strategy used Google Australia with English-language terms. The 2 independent searchers (RS, SH) were instructed to reset their cache in their Web browsers before each Google search to minimize the effect of Google search optimization. The 2 search term themes were “CVD/medication” and “risk.” The lead researchers (CB, LT) and the 2 independent searchers agreed upon 11 specific terms for CVD/medication (CVD, heart disease, stroke, heart attack, hypertension, hypercholesterolemia, hypercholesterolaemia, aspirin, blood pressure medication, cholesterol medication, and statin) and 2 specific terms for risk (risk calculator and risk assessment). A single CVD/medication and a single risk term were combined for a single search resulting in 22 unique Google searches. The first 50 results were considered (not including Web advertisements), providing a pool of 1100 results to be title scanned. The search results were limited to the first 50 after method piloting showed no additional websites would have been included up to 100 results. Searchers only recorded the Web addresses if they were to be assessed for eligibility. Duplicates were considered either as identical Web addresses, Web addresses that linked to the same risk calculator, or where a risk calculator from one webpage was embedded in another.

**Figure 1:**
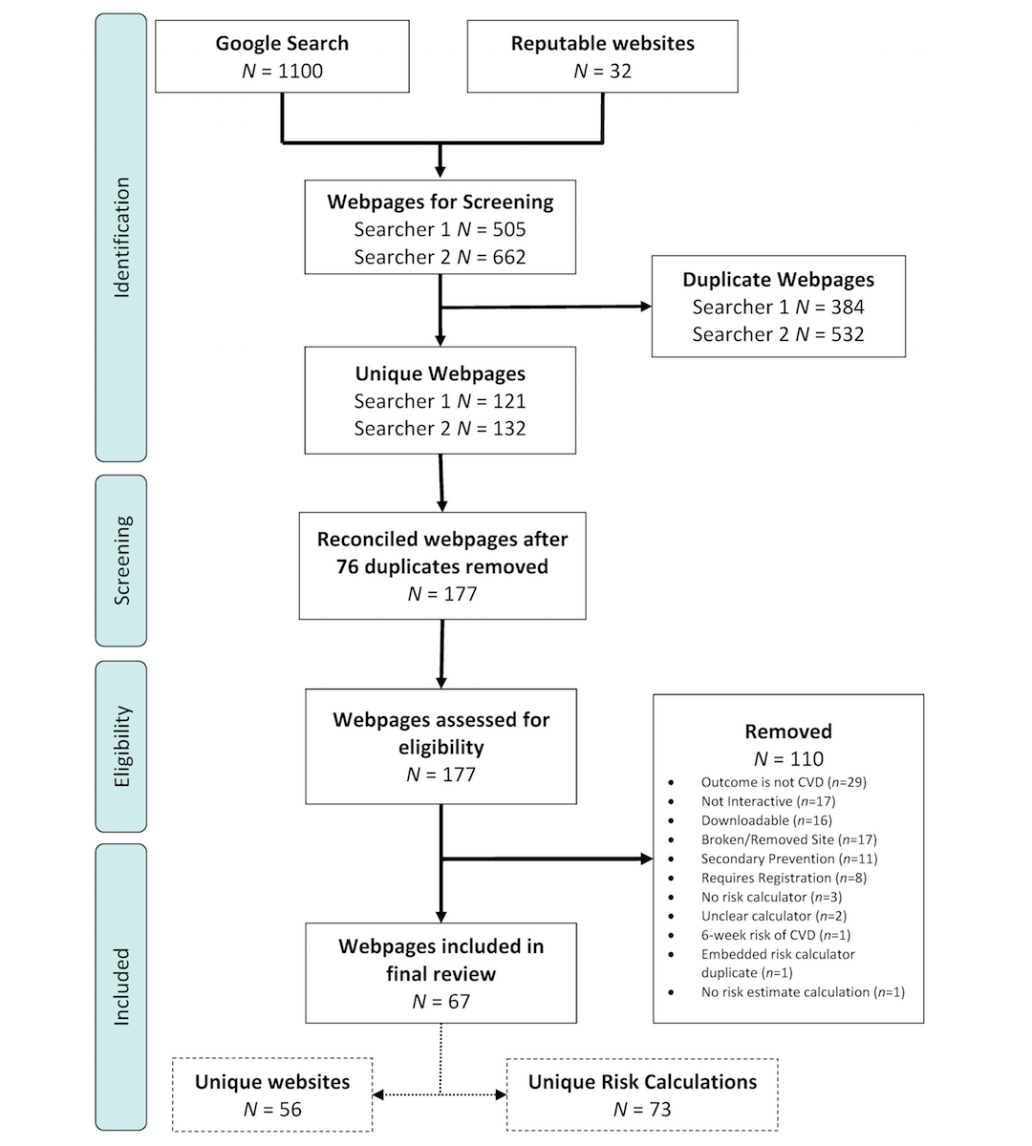
Search strategy and results (updated with higher res image).

The searchers (SH, RS) conducted this search as part of a Master of Public Health degree capstone unit from August to November 2016. In March 2017, an independent member of the team (MF) reconciled these search results based on the record of screened Web addresses provided by the original searchers (see Figure 1) by using Excel and removing duplicate Web addresses. The 2 independent searchers then rated risk calculators with the PEMAT-P providing 2 PEMAT-P ratings for 1 risk calculation.

### Evaluation and Data Extraction

The 2 searchers (SH, RS) developed the framework for basic data extraction by qualitatively describing the content of the risk calculator results for the high-risk profile. From this, a standard form was developed (CB, MF) to numerically record basic descriptive data for each calculator: risk model used, how the risk was presented (eg, relative risk, absolute risk, life expectancy, graphical formats) and recommended actions for the high-risk profile (eg, take medication, change lifestyle, see general practitioner). The third rater (MF) then extracted the data numerically, with any uncertainties discussed with the lead researcher (CB) to reach consensus.

The 2 searchers (SH, RS) also rated the content of each risk calculator using a validated tool, the PEMAT-P [27]. Searchers were instructed to first read the PEMAT-P user manual before proceeding with rating and spoke fortnightly with a supervising researcher (CB) to discuss/explain any items that were not immediately clear (eg, examples of an active voice). PEMAT-P provides 2 submeasures that are particularly relevant to health literacy: understandability, which is a measure of how well a health consumer is able to process and explain the key message of the material, where higher percentages indicate better understandability, and actionability, which is a measure of how well a health consumer is able to identify what to do based on the information presented, where higher percentages indicate better actionability. For the 2 independent searchers, the correlation between understandability scores was 0.57 and the correlation between actionability scores was 0.71. Discrepant item scores between the first 2 raters were resolved by a third rater (MF, after discussion with CB) to finalize the PEMAT-P score for each risk calculator. These decisions were double-checked with the original searchers, who agreed with the final approach.

A predefined high-risk cardiovascular profile for a hypothetical patient was used to assess the clinical validity of each calculator. This was a 65-year-old male smoker with systolic/diastolic blood pressure of 130/80 mm Hg, total/high-density lipoprotein cholesterol ratio of 6, and body mass index of 26 kg/m^2^. Where risk calculators had additional factors, the question was left blank (if possible) or an answer was given that either indicated the middle of the range or provided no additional risk on top of the risk profile (eg, a low-risk ethnicity, no history of CVD or taking medication). The 2 independent raters (SH, RS) created descriptive lists of the different clinical risk models, risk formats, and recommended actions for each calculator based on the high-risk profile. The third rater (MF) then used this framework to code the full dataset after discussion with CB, using the same high-risk profile.

## Results

### Search Results

This search yielded 67 unique webpages (see Figure 1). A list of the included Web addresses and their ID numbers can be found in Multimedia Appendix 1 (Table A). One website would sometimes host multiple risk calculators for different CVD events (eg, specifically for stroke or myocardial infarction). In total, these 67 webpages were found within 56 websites. Three calculators were able to calculate multiple absolute risks of future CVD events based on different models (ID2, ID4, ID10). These calculators were counted only once in the search strategy but are duplicated for the purposes of data extraction as different models of future CVD risk use different risk factors. Risk factor profiles are based on model-specific results. For example, ID2 is able to calculate risks based on 3 different models, therefore has been counted as 3 unique calculators. From the included 67 webpages, there are a total of 73 unique risk calculators.

### Risk Calculator Characteristics

The descriptive characteristics of the calculators are provided in Table 1.

The calculators used a variety of published risk models but the most common were those used in clinical practice guidelines: Framingham (44%), American College of Cardiology/American Heart Association (ACC/AHA) (10%), QRISK2 (5%), Reynolds (4%), and Assessing cardiovascular risk using the Scottish Intercollegiate Guidelines Network (ASSIGN) (3%). One-quarter did not specify the underlying model (25%). The outcomes included CVD/coronary heart disease (CHD), angina, heart attack/myocardial infarction, stroke, heart failure, kidney disease, diabetes, and CVD/CHD death. Most calculators used a 10-year time frame (77%) but some used 5-year (7%), 30-year (3%), or lifetime (7%) risk, and one allowed different estimates for the range of 1 to 10 years (1%). Many risk calculators did not state specific outcomes beyond mentioning CVD risk, and some did not state the time frame.

The absolute percentage risk was stated in most but not all calculators (79%). Other risk formats included categorical risk with 2 to 4 groups ranging from low to high (32%), a verbal description of the frequency such as 8 in 100 (18%), heart age (10%), life expectancy (3%), and relative risk (3%). In 34 risk calculators (47%) only 1 risk format was presented, and in 39 (53%) risk calculators 2 to 5 risk formats were presented. The risk profile outlined previously yielded highly variable results depending on the model and outcomes used, with an absolute risk ranging from 3% to 43% over 10 years, a heart age of 68 to 86 years, a life expectancy of 79 to 84 years, and a relative risk of 1.8 to 2.1 compared to a healthy person’s risk. Visual aids included icon arrays or pictographs (18%), bar or line graphs (16%), and charts showing risk level (10%).

**Figure 2:**
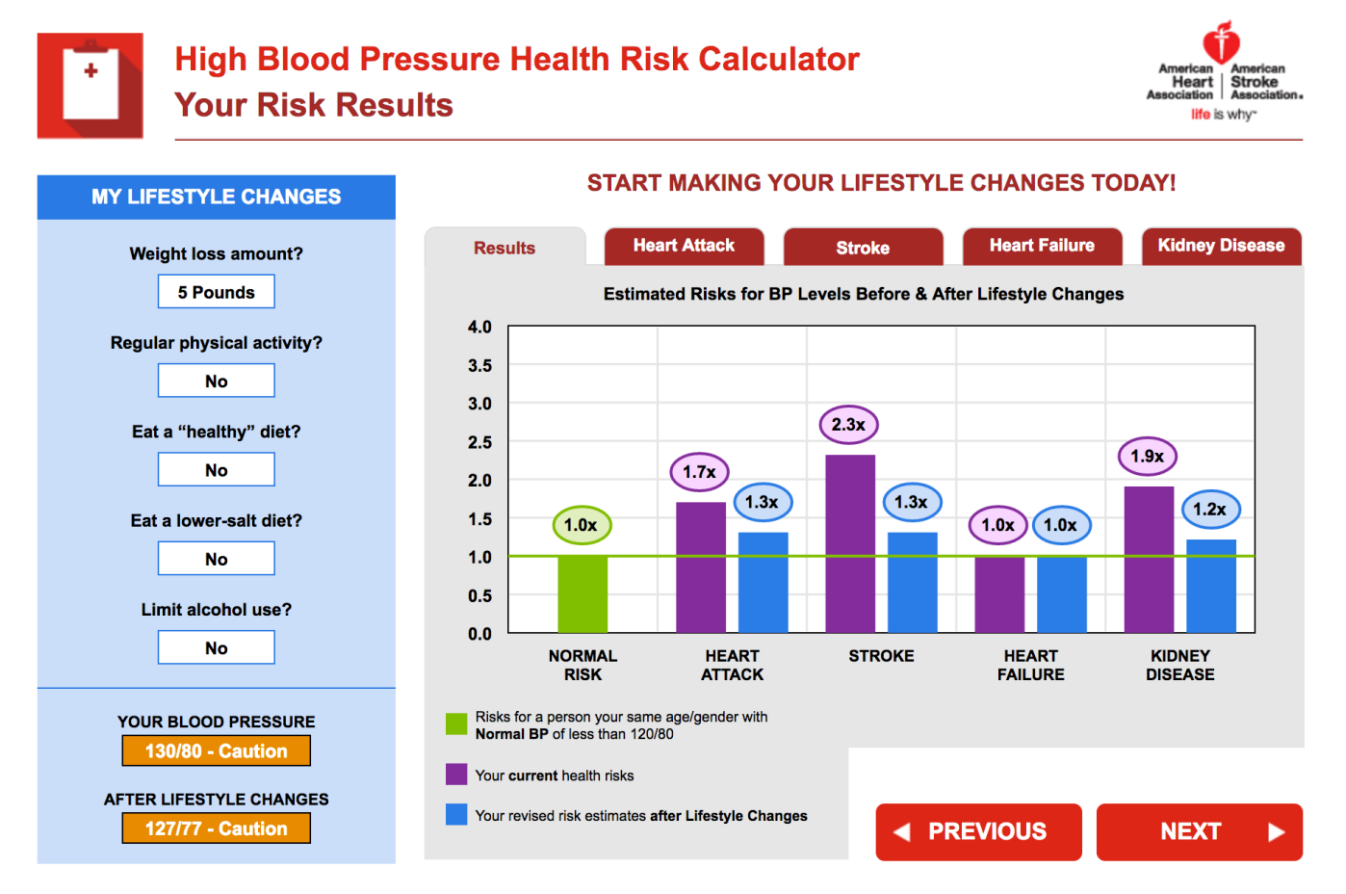
An example of a risk calculator with a high understandability Patient Education Material Evaluation Tool for Print Materials score.

**Figure 3:**
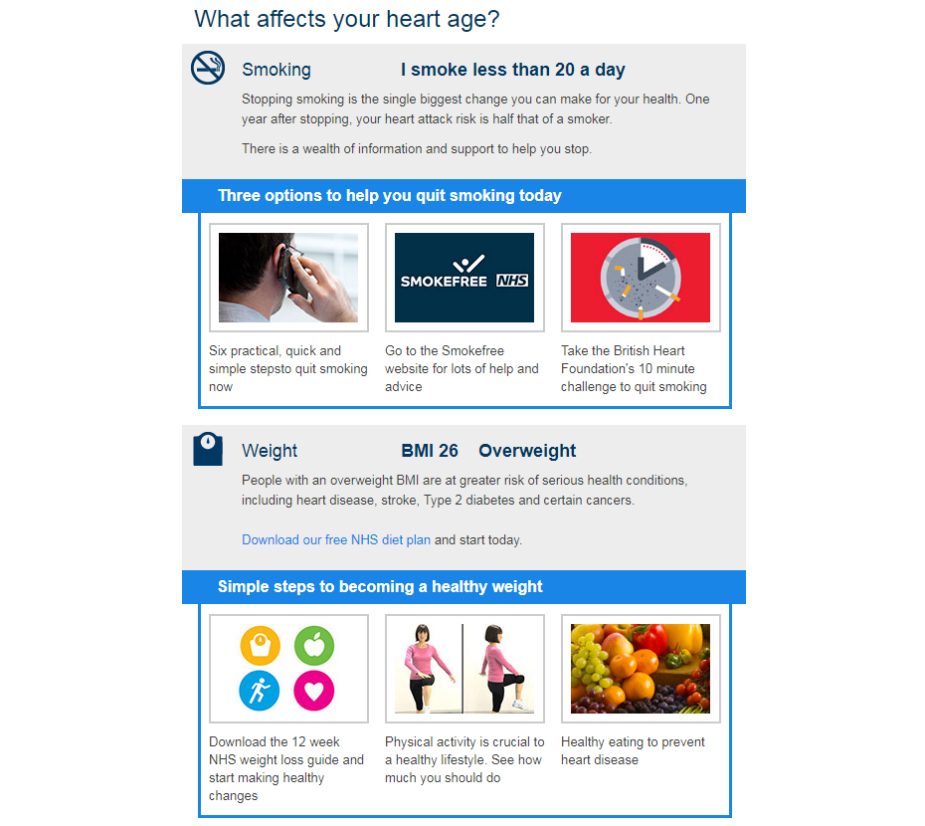
An example of a risk calculator with a high actionability Patient Education Material Evaluation Tool for Print Materials score.

For the PEMAT-P evaluation, the calculators scored moderately on understandability and poorly on actionability. The average understandability score was 64% (SD 20%) which ranged from 30% to 100%, and the average actionability score was 19% (SD 26%) which ranged from 0 to 100%. Screenshots from very high-scoring examples for understandability (ID14) and actionability (ID66) are shown in Figures 2 and 3, respectively. Reliability was variable for the individual PEMAT-P items, with Cohen kappa scores ranging from –0.05 to .65 and agreement ranging from 42% to 99%. These discrepancies were resolved by using a third rater and team discussion to reach consensus on each decision. The PEMAT-P scores for individual risk calculators and reliability by PEMAT-P item are provided in Multimedia Appendix 1 (Tables B and C).

**Table 1:** Characteristics of final calculators (n=73).

Characteristic		Count, n (%)
**Risk model**		
	Framingham	32 (44)
	Not stated	18 (25)
	ACC/AHA^a^	7 (10)
	QRISK2	4 (5)
	Reynolds Risk Score	3 (4)
	ASSIGN^b^	2 (3)
	ARIC^c^ Study	1 (1)
	BNF^d^	1 (1)
	Health Professional Follow-Up Study and Nurses’ Health Study	1 (1)
	MESA^e^	1 (1)
	Pocock et al (2001)	1 (1)
	QStroke	1 (1)
	Strong Heart Study	1 (1)
**Risk format**		
	Absolute risk	58 (79)
	Categorical risk	23 (32)
	Frequency	13 (18)
	Heart age	7 (10)
	Life expectancy	2 (3)
	Relative risk	2 (3)
	Icon array/pictograph	13 (18)
	Graphs	12 (16)
	Charts	7 (10)
**Recommended actions**		
	Stop smoking	21 (29)
	Lower cholesterol/take cholesterol medication	21 (29)
	Lower blood pressure/take blood pressure medication	14 (19)
	Improve diet	10 (14)
	Increase physical activity	9 (12)
	Seek doctor’s advice	9 (12)
	Take aspirin	6 (8)
	Address body mass index	3 (4)

^a^ACC/AHA: American College of Cardiology/American Heart Association.

^b^ASSIGN: Assessing cardiovascular risk using the Scottish Intercollegiate Guidelines Network.

^c^ARIC: Atherosclerosis Risk in Communities.

^d^BNF: British National Formulary.

^e^MESA: Multi-Ethnic Study of Atherosclerosis.

## Discussion

### Principal Findings

This review found 73 unique CVD risk calculators available to the public online that use a wide variety of risk models, risk communication formats, understandability, and actionability. Of particular concern is the variation in CVD risk estimates based on entering the same hypothetical high-risk patient data with little explanation for why this would occur: ranging from as little as 3% to as high as a 43% risk of a CVD event over the next 10 years. One-quarter (25%) of the risk calculators did not specify the underlying model, study, calculations, or assumptions, so it not possible to assess their validity or the reasons for variation. The remaining three-quarters (75%) did specify enough information to determine the underlying algorithm, where differences in the study population (eg, US-based Framingham versus UK-based QRISK), included risk factors (eg, whether less directly predictive factors like body mass index were included as well as strong clinical indicators like cholesterol), and CVD outcomes (eg, mortality only, nonfatal heart attack and stroke, angina) explain the discrepant risk results for the same profile. In the academic literature, there are over 17 validated CVD risk models [19], and both the United Kingdom and United States have moved on from the original 10-year Framingham model in their current clinical guidelines [13,15]. In contrast, Framingham is still the dominant model online, but there were no publicly available 5-year risk calculators that scored very highly on both understandability and actionability. This means that countries like Australia that use a 5-year Framingham model could benefit from additional development using highly rated features of 10-year calculators (Figures 2 and 3). Patients with low health literacy searching for their own CVD risk information are most likely to encounter 10-year models that do not correspond to local clinical guidelines.

In terms of risk communication, absolute risk should ideally be presented in a variety of formats to cater to different needs and learning styles [28]. While absolute risk, verbal explanations of risk categories, and graphical presentations were all present in our sample of risk calculators, just over half used a combination of different presentation formats for the same numerical information. At minimum, all calculators should present the absolute risk in numerical form, but over 20% did not meet this minimum standard. Icon arrays displaying risk in terms of CVD event frequencies were only used in 18% of risk calculators, despite much attention in the risk communication literature and recommendations to use these formats in international patient decision aid standards [28].

The PEMAT-P evaluation was chosen for its unique focus on reducing health literacy demand through both understandability and actionability [29]. It does not yet have an agreed threshold for acceptable levels, but comparison to recent studies of information for related conditions suggests that understandability was moderate and actionability was poor overall (present study: 64% and 19%; online heart failure websites: 56% and 35%; printed lifestyle information for chronic kidney disease: 52% and 37% for understandability and actionability, respectively) [30,31]. To improve actionability, clinical guidelines recommend both lifestyle and medication for the selected high-risk patient profile [12], but even the most strongly recommended action of quitting smoking was only mentioned by 29% of calculators, and there was more focus on statins than blood pressure–lowering medication even though both risk factors were elevated compared to ideal levels. Presenting clear, jargon-free information on all the available options as well as referring the user to a doctor to discuss high-risk results is recommended to better meet the needs of low health literacy users.

The findings of this study are comparable to broader literature on the quality of online health information; a review showing 70% of studies evaluating 5941 websites concluded that higher quality is needed [10]. For a recent example, the US Department of Health and Human Services’ Office of Disease Prevention and Health Promotion evaluated the quality of the 100 top-ranked health-related websites and found that only 58% met at least 3 out of 6 reliability criteria, while 42% followed at least 10 out of 19 usability principles [32]. This was part of an effort to set national objectives to improve the quality of eHealth by 2020, to which health literacy criteria could perhaps be added in future.

### Strengths and Limitations

The strengths of this study include a systematic review and evaluation process with multiple independent searchers/raters. The main limitation is the replicability of conducting a systematic search using online search engines like Google. The dynamic nature of the Web with constant variation in website content and metadata means that no search is perfectly replicable even though the cache was cleared between search terms. However, the methods used are likely to have captured the most common and popular search results, since many duplicates were removed between the 2 searchers. It is likely that additional calculators existed at the time of the search and could potentially have been found by a different searcher, search engine, or geographical location, but this study provides a comprehensive list of accessible calculators at the time of searching.

The variable reliability of the PEMAT-P items was slightly lower than previous research [33], but could possibly be improved by recoding the low-scoring items or through further training of the coders, although the PEMAT-P developers intended it for use by nonexperts [29]. Lower PEMAT-P reliability could also be due to the risk calculators not fitting PEMAT definitions for printable materials. Reevaluation was not possible in this study due to the changing nature of the interactive risk calculator websites but could be considered for future research using PEMAT-P with static materials. The data extraction for basic descriptive content (risk model and risk result formats) was conducted by an individual researcher in this study, so reliability could not be assessed, although this is not generally reported in systematic review data extraction [26].

### Implications

The plethora of calculators and wide variation in results from the same input have the potential to confuse and harm the general public if appropriate medical advice is not sought. Actionability scores are poor on average, and minimum risk communication standards are not being met. Future research evaluating the suitability of online risk calculators for low health literacy users would benefit from a revised version of the PEMAT-P designed specifically for interactive online formats. Existing items relevant to health literacy may need to be clarified, including (1) better definition for the presence/absence of a table, since website content is often built around a table format, (2) instructions for dealing with interactive graphs based on buttons, (3) a definition of short material for webpages, (4) how to address subtle visual aids such as color, size, or positioning which are more heterogeneous in interactive online formats, and (5) how to best address actionability items with general health advice versus personalized advice based on the risk result. In addition, concepts from eHealth evaluation tools could be incorporated, including (1) ease of navigation through the calculator; (2) presence/absence of distractions in the webpage (eg, advertisements, pop-ups); and (3) ability to save, print, or email the results and recommended actions. Finally, additional information is needed to enable clinicians and consumers to determine whether health risk calculators give a reliable estimate, including the model or algorithm used (eg, study name/reference, outcomes measured, and time frame), what population the calculation has been validated in (eg, age, sex, and ethnicity), an explanation of how this relates to current clinical guidelines, and when the evidence for the calculation was last updated.

### Conclusion

Online CVD risk calculators produce highly variable results for the same person with little explanation for why this would occur. Differences in the models used, risk factors included, risk communication formats presented, and actions specified explain part of this variation, but one-quarter of risk calculators did not specify any underlying assumptions. Health professionals should be aware of the reasons for conflicting results that patients might encounter, and developers need to address actionability as well as clinical validity and understandability to improve usefulness to the majority of the population with low health literacy. Country-specific calculators that match national clinical guidelines and build on examples with high understandability and actionability scores would benefit both health professionals and consumers.

## Appendix

Multimedia Appendix 1 Risk calculator archive and Patient Education Material Evaluation Tool for Print Materials scores.

## Abbreviations

ACC: American College of Cardiology
AHA: American Heart Association
ARIC: Atherosclerosis Risk in Communities
ASSIGN: Assessing cardiovascular risk using the Scottish Intercollegiate Guidelines Network
BNF: British National Formulary
CHD: coronary heart disease
CVD: cardiovascular disease
MESA: Multi-Ethnic Study of Atherosclerosis
PEMAT-P: Patient Education Material Evaluation Tool for Print Materials
