# Supplementary material for: Clinical Validity, Understandability, and Actionability of Online Cardiovascular Disease Risk Calculators: Systematic Review
Source: J Med Internet Res. 2018 Feb 1;20(2):e29. doi: 10.2196/jmir.8538 (PMC5814602; doi:10.2196/jmir.8538)
Supplement: Multimedia Appendix 1 [file jmir_v20i2e29_app1.pdf]

Multimedia Appendix 1: Risk Calculator Archive and PEMAT-P Scores

Table A: Web archive of included Cardiovascular Disease Risk Calculators

ID	Web address	WebCite
1	http://aricnews.net/riskcalc/html/rc1.html	http://www.webcitation.org/6rB7aUByj
2	http://chd.bestsciencemedicine.com/calc2.html	http://www.webcitation.org/6rB8ql4nD
3	http://clincalc.com/cardiology/ascvd/pooledcohort.aspx	http://www.webcitation.org/6rB7l3O59
4	http://cvrisk.mvm.ed.ac.uk/calculator/calc.asp	http://www.webcitation.org/6rB7gh6nt
5	http://healthtools.bodyandhealth.canada.com/health_tools.asp?t=82&text_id=4720&channel_id=2172&relation_id=112942	http://www.webcitation.org/6rB637EWc
6	http://patient.info/doctor/cardiovascular-risk-calculator	http://www.webcitation.org/6rB9MTrC6
7	http://reference.medscape.com/calculator/aac-aha-cardiovascular-risk-ascvd	http://www.webcitation.org/6rB8vTlH2
8	http://reference.medscape.com/calculator/framingham-cardiovascular-disease-risk	http://www.webcitation.org/6rB9UeaN4
9	http://reference.medscape.com/calculator/framingham-coronary-risk-cholesterol	http://www.webcitation.org/6rB7rcwN8
10	http://statindecisionaid.mayoclinic.org/index.php/statin/index	http://www.webcitation.org/6rB7TxoW4
11	http://stroke.ucla.edu/stroke-risk-calculator	http://www.webcitation.org/6rB6yZjuM
12	http://strongheart.ouhsc.edu/CHDcalculator/calculator.html	http://www.webcitation.org/6rB9cqk6X
13	http://tools.acc.org/ASCVD-Risk-Estimator/	http://www.webcitation.org/6rB8TqNFr
14	http://tools.bigbeelabs.com/aha/tools/hbp/	http://www.webcitation.org/6rB5r8B4H
15	http://www.assign-score.com/estimate-the-risk/	http://www.webcitation.org/6rB9h3yHq
16	http://www.bhgp.co.uk/cardiocalc.asp	http://www.webcitation.org/6rB9vfUrP
17	http://www.bshp.edu/Files/Baptist/Flash/StrokeRiskCalculator/index.htm	http://www.webcitation.org/6rB5wauRR
18	http://www.cdc.gov/vitalsigns/cardiovasculardisease/heartage.html	http://www.webcitation.org/6rB9znEPE
19	http://www.cimedicalcenter.com/heart-attack-calculator-p126	http://www.webcitation.org/6rBA3ilNz
20	http://www.cvdcheck.org.au/	http://www.webcitation.org/6rBA9coEd
21	http://www.cvriskcalculator.com/	http://www.webcitation.org/6rB8ejEoB
22	http://www.framinghamheartstudy.org/risk-functions/cardiovascular-disease/10-year-risk.php#	http://www.webcitation.org/6rBAD0e2U
23	http://www.framinghamheartstudy.org/risk-functions/cardiovascular-disease/30-year-risk.php#	http://www.webcitation.org/6rBAHRMEZ
24	http://www.globalrph.com/pooled.htm	http://www.webcitation.org/6rB5kcQJV
25	http://www.heart.org/gglRisk/locale/en_US/index.html?gtype=health	http://www.webcitation.org/6rB8jT1NQ
26	http://www.knowyournumbers.co.nz/heart-age-forecast.aspx	http://www.webcitation.org/6rBAL6AtE
27	http://www.mayoclinic.org/heart-disease-risk/itt-20084942	http://www.webcitation.org/6s0gr0XJU
28	http://www.mcw.edu/calculators/Coronary-Heart-Disease-Risk.htm	http://www.webcitation.org/6rBDxzJRb
29	http://www.mcw.edu/calculators/LDL-Cholesterol-Goal-Level.htm	http://www.webcitation.org/6rBE0IwwL

30	http://www.mdcalc.com/framingham-coronary-heart-disease-risk-score/	http://www.webcitation.org/6rB83owdW
31	http://www.medcalc.com/heartrisk.html	http://www.webcitation.org/6rBE3hbO3
32	http://www.medindia.net/patients/calculators/cardiacrisk.asp	http://www.webcitation.org/6rBEBolwf
33	http://www.medindia.net/patients/calculators/stroke-risk-calculator.asp	http://www.webcitation.org/6rB7BeOPk
34	http://www.mountcarmelhealth.com/fast	http://www.webcitation.org/6rBEeKzcR
35	http://www.mydr.com.au/tools/heart-disease-risk-test	http://www.webcitation.org/6rB89RHQu
36	http://www.mydr.com.au/tools/stroke-risk-test	http://www.webcitation.org/6rB69ujlJ
37	http://www.ohsu.edu/xd/health/services/brain/getting-treatment/diagnosis/stroke/stroke-risk-assessment-test.cfm	http://www.webcitation.org/6rB6GXNoV
38	http://www.premierhealth.com/Your-Wellness/Live-Well-Stay-Well/Know-Your-Risk/Heart-Risk-Assessment/	http://www.webcitation.org/6rBEh5yOf
39	http://www.projectbiglife.ca/life/index.php?le=1&fhc=0&str=1	http://www.webcitation.org/6rBEl1Nsl
40	http://www.qintervention.org/	http://www.webcitation.org/6rBEpbUjd
41	http://www.qstroke.org/	http://www.webcitation.org/6rAwDbbCE
42	http://www.reynoldsriskscore.org/	http://www.webcitation.org/6rB8PZtLD
43	http://www.riskscore.org.uk/	http://www.webcitation.org/6rBF6Ua5G
44	http://www.vizhealth.org/using/calculator/	http://www.webcitation.org/6rBF9V0gR
45	http://www.womensheart.org/content/heartdisease/heart_disease_risk_quiz.asp	http://www.webcitation.org/6rBFDFtOW
46	http://www.yourdiseaserisk.wustl.edu/YDRDefault.aspx?ScreenControl=YDRGeneral&ScreenName=YDRHeart	http://www.webcitation.org/6rBFGPEjw
47	http://www.yourdiseaserisk.wustl.edu/YDRDefault.aspx?ScreenControl=YDRGeneral&ScreenName=YDRStroke	http://www.webcitation.org/6rBFJziW8
48	http://www.zunis.org/Framingham%20Risk%20of%20CHD2.htm	http://www.webcitation.org/6rBFOhsvn
49	https://ehealth.heartandstroke.ca/	http://www.webcitation.org/6rBFSVabq
50	https://healthyheartscore.sph.harvard.edu/	http://www.webcitation.org/6rBFWyfBN
51	https://mhgpc.com/risk-calculator.html	http://www.webcitation.org/6rB8KPVQn
52	https://myhealth.alberta.ca/Alberta/Pages/Heart-Disease-Risk-Calculator.aspx	http://www.webcitation.org/6rBFa9Xa0
53	https://qrisk.org/2015/	http://www.webcitation.org/6rBFdpUo2
54	https://qrisk.org/2016/	http://www.webcitation.org/6rBFgBWEX
55	https://qrisk.org/lifetime/	http://www.webcitation.org/6rBFjIZUn
56	https://softwarecorp.es/asariskcalculator/public/calculator/en	http://www.webcitation.org/6rBFpIY1U
57	https://www.acefitness.org/acefit/healthy_living_tools_content.aspx?id=10	http://www.webcitation.org/6rBFtIo54
58	https://www.cardiosmart.org/tools/heart-disease-risk-assessment	http://www.webcitation.org/6rBFxcX33
59	https://www.cvdriskchecksecure.com/framinghamriskscore.aspx	http://www.webcitation.org/6rBG2AL9H
60	https://www.cvdriskchecksecure.com/ReynoldsRiskScore.aspx	http://www.webcitation.org/6rBG4Ovnd
61	https://www.dileyridgemedicalcenter.com/patient-information/health-information/heart-attack-risk-assessment	http://www.webcitation.org/6rBG7PqDz
62	https://www.ghc.org/html/public/tools/heart/	http://www.webcitation.org/6rBGEj6Vl
63	https://www.med-decisions.com/h2hv2/	http://www.webcitation.org/6rBGRO6iB

64	https://www.mesa-nhlbi.org/MESACHDRisk/MesaRiskScore/RiskScore.aspx	http://www.webcitation.org/6rB7QdI0p
65	https://www.mygoodhealth.ca/healthtools/health_tools.asp?t=17&text_id=2704&n=Heart+disease+risk+calculator	http://www.webcitation.org/6rBGXGFPf
66	https://www.nhs.uk/Tools/Pages/heartage.aspx	http://www.webcitation.org/6rBGsY4MB
67	https://www.uptodate.com/contents/calculator-cardiovascular-risk-assessment-10-year-men-patient-education	http://www.webcitation.org/6rBGpAxFq

Table B. PEMAT-P Understandability and Actionability Scores

ID	Web address	Understandability	Actionability
1	http://aricnews.net/riskcalc/html/rc1.html	55%	0%
2	http://chd.bestsciencemedicine.com/calc2.html	62%	0%
3	http://clincalc.com/cardiology/ascvd/pooledcohort.aspx	69%	17%
4	http://cvrisk.mvm.ed.ac.uk/calculator/calc.asp	33%	0%
5	http://healthtools.bodyandhealth.canada.com/health_tools.asp?t=82&text_id=4720&channel_id=2172&relation_id=112942	80%	20%
6	http://patient.info/doctor/cardiovascular-risk-calculator	40%	0%
7	http://reference.medscape.com/calculator/aac-aha-cardiovascular-risk-ascvd	30%	0%
8	http://reference.medscape.com/calculator/framingham-cardiovascular-disease-risk	50%	0%
9	http://reference.medscape.com/calculator/framingham-coronary-risk-cholesterol	40%	0%
10	http://statindecisionaid.mayoclinic.org/index.php/statin/index	77%	50%
11	http://stroke.ucla.edu/stroke-risk-calculator	50%	0%
12	http://strongheart.ouhsc.edu/CHDcalculator/calculator.html	50%	0%
13	http://tools.acc.org/ASCVD-Risk-Estimator/	60%	20%
14	http://tools.bigbeelabs.com/aha/tools/hbp/	100%	33%
15	http://www.assign-score.com/estimate-the-risk/	40%	0%
16	http://www.bhgp.co.uk/cardiocalc.asp	75%	80%
17	http://www.bshp.edu/Files/Baptist/Flash/StrokeRiskCalculator/index.htm	80%	0%
18	http://www.cdc.gov/vitalsigns/cardiovasculardisease/heartage.html	50%	0%
19	http://www.cimedicalcenter.com/heart-attack-calculator-p126	62%	0%
20	http://www.cvdcheck.org.au/	85%	40%
21	http://www.cvriskcalculator.com/	50%	60%
22	http://www.framinghamheartstudy.org/risk-functions/cardiovascular-disease/10-year-risk.php#	40%	0%
23	http://www.framinghamheartstudy.org/risk-functions/cardiovascular-disease/30-year-risk.php#	40%	0%
24	http://www.globalrph.com/pooled.htm	50%	0%
25	http://www.heart.org/gglRisk/locale/en_US/index.html?gtype=health	88%	80%
26	http://www.knowyournumbers.co.nz/heart-age-forecast.aspx	77%	33%

27	http://www.mayoclinic.org/heart-disease-risk/itt-20084942	90%	60%
28	http://www.mcw.edu/calculators/Coronary-Heart-Disease-Risk.htm	40%	0%
29	http://www.mcw.edu/calculators/LDL-Cholesterol-Goal-Level.htm	30%	0%
30	http://www.mdcalc.com/framingham-coronary-heart-disease-risk-score/	50%	0%
31	http://www.medcalc.com/heartrisk.html	40%	0%
32	http://www.medindia.net/patients/calculators/cardiacrisk.asp	70%	60%
33	http://www.medindia.net/patients/calculators/stroke-risk-calculator.asp	90%	60%
34	http://www.mountcarmelhealth.com/fast	80%	0%
35	http://www.mydr.com.au/tools/heart-disease-risk-test	50%	0%
36	http://www.mydr.com.au/tools/stroke-risk-test	80%	20%
37	http://www.ohsu.edu/xd/health/services/brain/getting-treatment/diagnosis/stroke/stroke-risk-assessment-test.cfm	80%	20%
38	http://www.premierhealth.com/Your-Wellness/Live-Well-Stay-Well/Know-Your-Risk/Heart-Risk-Assessment/	78%	40%
39	http://www.projectbiglife.ca/life/index.php?le=1&fhc=0&str=1	70%	0%
40	http://www.qintervention.org/	69%	40%
41	http://www.qstroke.org/	69%	0%
42	http://www.reynoldsriskscore.org/	62%	0%
43	http://www.riskscore.org.uk/	62%	0%
44	http://www.vizhealth.org/using/calculator/	85%	40%
45	http://www.womensheart.org/content/heartdisease/heart_disease_risk_quiz.asp	67%	40%
46	http://www.yourdiseaserisk.wustl.edu/YDRDefault.aspx?ScreenControl=YDRGeneral&ScreenName=YDRHeart	93%	50%
47	http://www.yourdiseaserisk.wustl.edu/YDRDefault.aspx?ScreenControl=YDRGeneral&ScreenName=YDRStroke	93%	33%
48	http://www.zunis.org/Framingham%20Risk%20of%20CHD2.htm	50%	0%
49	https://ehealth.heartandstroke.ca/	92%	40%
50	https://healthyheartscore.sph.harvard.edu/	91%	80%
51	https://mhgpc.com/risk-calculator.html	50%	0%
52	https://myhealth.alberta.ca/Alberta/Pages/Heart-Disease-Risk-Calculator.aspx	85%	0%
53	https://qrisk.org/2015/	85%	17%
54	https://qrisk.org/2016/	85%	17%
55	https://qrisk.org/lifetime/	62%	17%
56	https://softwarecorp.es/asariskcalculator/public/calculator/en	33%	20%
57	https://www.acefitness.org/acefit/healthy_living_tools_content.aspx?id=10	92%	0%
58	https://www.cardiosmart.org/tools/heart-disease-risk-assessment	60%	40%
59	https://www.cvdriskchecksecure.com/framinghamriskscore.aspx	40%	20%
60	https://www.cvdriskchecksecure.com/ReynoldsRiskScore.aspx	50%	0%

61	https://www.dileyridgemedicalcenter.com/patient-information/health-information/heart-attack-risk-assessment	78%	0%
62	https://www.ghc.org/html/public/tools/heart/	82%	60%
63	https://www.med-decisions.com/h2hv2/	71%	0%
64	https://www.mesa-nhlbi.org/MESACHDRisk/MesaRiskScore/RiskScore.aspx	30%	0%
65	https://www.mygoodhealth.ca/healthtools/health_tools.asp?t=17&text_id=2704&n=Heart+disease+risk+calculator	80%	0%
66	https://www.nhs.uk/Tools/Pages/heartage.aspx	94%	100%
67	https://www.uptodate.com/contents/calculator-cardiovascular-risk-assessment-10-year-men-patient-education	60%	0%

Table C. Inter-rater agreement between two raters for each PEMAT-P Item

PEMAT-P item	Inter-rater agreement (%)	Cohen's Kappa
Understandability		
Content		
1. The material makes its purpose completely evident	55	.08
2. The material does not include information or content that distracts from its purpose	93	-.05
Word Choice and Style		
3. The material uses common, everyday language	72	.38
4. Medical terms are used only to familiarize audience with terms. When used, medical terms are defined	75	.48
5. The material uses the active voice	55	.20
Use of Numbers		
6. Numbers appearing in the material are clear and easy to understand	60	.37
7. The material does not expect the user to perform calculations	96	-.01
Organisation		
8. The material breaks of "chunks" information into short sections	84	.18
9. The material's sections have informative headers	84	.16
10. The material presents information in a logical sequence	82	-.04
11. The material provides a summary	42	.05
Layout and Design		
12. The material uses visual cues (e.g. arrows, boxes, bullets, bold, larger font, highlighting) to draw attention to key points	75	.42
Use of Visual Aids		
15. The material uses visual aids whenever they could make content more easily understood (e.g. illustration of healthy portion size)	62	.17
16. The material's visual aids reinforce rather than distract from the content	73	.47
17. The material's visual aids have clear titles or captions	67	.39
18. The material uses illustration and photographs that are clear and uncluttered	76	.55
19. The material uses simple tables with short and clear row and column headings	64	.01
Actionability		
20. The material clearly identified at least one action the user can take	81	.65
21. The material addresses the user directly when describing actions	81	.60
22. The material breaks down any action into manageable, explicit steps	88	.57
23. The material provides a tangible tool (e.g. menu planners, checklists) whenever it could help the use take action	99	.65
24. The material provides simple instructions or examples of how to perform calculations	99	-[a]
25. The material explains how to use the charts, graphs, tables, or diagrams to take actions	82	-[a]
26. The material uses visual aids whenever they could make it easier to act on the instructions	93	.27

[a]Unable to calculate as one rater had no variability
